# Assessing the population structure of *Plagioscion squamosissimus* (Teleostei, Perciformes, Sciaenidae) from the São Francisco River, Bahia, Brazil, using body morphology and otolith shape signatures

**DOI:** 10.1111/jfb.70221

**Published:** 2025-09-11

**Authors:** F. L. Freitas, N. S. Pereira, P. B. Pinheiro, R. Schroeder, A. T. Correia

**Affiliations:** ^1^ Department of Technology and Social Science State University of Bahia Juazeiro Brazil; ^2^ Department of Exact and Earth Science State University of Bahia Salvador Brazil; ^3^ Department of Education State University of Bahia Paulo Afonso Brazil; ^4^ Laboratory of Marine Applied Studies, Polytechnic School University of Vale do Itajaí Itajaí Brazil; ^5^ Interdisciplinary Centre of Marine and Environmental Research, Terminal de Cruzeiros do Porto de Leixões Matosinhos Portugal; ^6^ Department of Aquatic Production (DPA), School of Medicine and Biomedical Sciences (ICBAS) University of Porto (UP) Porto Portugal

**Keywords:** elliptic Fourier descriptors, fish stocks, natural tags, Procrustes coordinates, Sciaenidae, truss networking, wavelet transform coefficients

## Abstract

The south American silver croaker, *Plagioscion squamosissimus*, holds significant importance for the artisanal fisheries in the sub‐middle and lower courses of the São Francisco River, located in northeastern Brazil. To investigate the population structure of this species along its longitudinal profile, a total of 150 individuals (50 fish per site), measuring between 35 and 45 cm in standard length, were collected from three fishers' associations (Olhos D'água do Casado, Petrolândia and Rodelas) using gill nets from September 2023 to March 2024. Morphometry analyses of body shape were conducted using two landmark‐based geometric morphometrics: Procrustes coordinates (PCs) and truss networking (TD). Additionally, otolith shape was examined through two otolith contour analyses: wavelet transform coefficients (WTCs) and elliptic Fourier descriptors (EFDs). Both univariate and multivariate statistical approaches were employed to assess the data. The findings revealed a significant secondary sexual dimorphism in otolith shape that was not observed in body shape. This dimorphism may be linked to sex‐specific hearing adaptations associated with ecological or reproductive behavioural strategies, although further research is required to test this hypothesis. Despite this sexual dimorphism, the overall reclassification success rate of individuals to their original sites was notably high, ranging from 91% to 94% using body morphometry (TD and PCs, respectively), and reaching 100% accuracy with otolith shape analysis (EFDs and WTCs). The phenotypic differences observed among local populations likely resulted from distinct environmental conditions between sites, probably caused by the construction of two hydroelectric plants that disrupted fish connectivity along the São Francisco River. The results suggest that *P. squamosissimus* populations captured in the sub‐middle and lower courses of the river constitute distinct population‐units. This distinction has critical implications for the species' management and conservation strategies.

## 1 INTRODUCTION

The distribution and population dynamics of fish within aquatic environments are influenced not only by abiotic factors, such as temperature, salinity and depth (Brooker et al., 2020; Costa et al., 2022; Ferreira et al., 2024), but also by biotic factors, including food availability and refuge from predators (Ferreira et al., 2024; Franssen et al., 2013; Wright et al., 2020). In freshwater ecosystems, dam construction alters the physical and chemical characteristics of rivers, impacting both the abundance and composition of fish communities (Agostinho et al., 2016; Carvajal‐Quintero et al., 2017; Winemiller et al., 2016). Such changes can lead to the extreme proliferation of some species or populations, while others may experience a decline in abundance or even local extinction (Agostinho et al., 2008; Nilsson et al., 2005; Simonovic et al., 2021). Identifying the structure of fish stocks and delineating their geographic boundaries are essential elements for a rational and sustainable fisheries management (Cadrin et al., 2014).

The Sciaenidae family is predominantly composed of marine and estuarine fish species; however, four strictly freshwater genera are found in South America, including the South American silver croaker, *Plagioscion squamosiss*imus (Casatti, 2005), which is native to the Orinoco, Amazon and Guiana River basins. It has been introduced into the Paraná‐Paraguay‐Uruguay river basins and the São Francisco River basin since the 1950s (Agostinho et al., 2007; Casatti, 2005). Under non‐anoxic conditions, *P. squamosissimus* demonstrates strong adaptation to the demersal environments typical of freshwater reservoirs (Agostinho et al., 2007). The species exhibits a continuous reproductive cycle with seasonal spawning peaks throughout the year (Queiroz‐Sousa et al., 2018) and reaches first maturity at a length of 15.9 cm in the Santa Cruz Reservoir (Sousa et al., 2015) and 19.6 cm in the lower Amazon River (Lima et al., 2019). Individuals are opportunistic carnivores, feeding on zooplankton, shrimps and fish (Bennemann et al., 2006; Hahn et al., 2008). According to fisheries statistics, the global annual catch of this species was 17,900 tons in 2022 (FAO, 2024).

The São Francisco River in Brazil is recognized as a major contributor to the national freshwater fish production, serving as a vital source of fish for markets in the northeastern and southeastern regions of the country (Campeche et al., 2011; Godinho & Godinho, 2003; Sato & Godinho, 2003). It the1980s, an estimated of 26,000 fishers operated within the São Francisco River basin, increasing to 34,244 by the mid‐2000s (Sato & Godinho, 2003; Tomáz & Marques, 2015). Although comprehensive national statistics on current fish landings are lacking, available evidence suggests small‐scale fisheries have declined in recent years in northeastern Brazil (Seminara et al., 2024). This reduction has been attributed to multiple factors, including anthropogenic pollution, unsustainable land use, inadequate fisheries legislation, overexploitation and river fragmentation caused by the construction of numerous dams along the river's course (Agostinho et al., 2008).

The São Francisco River is approximately 2914 km (about 1811 miles) long, making it one of the longest rivers in Brazil and South America. It flows from its source in the southeastern state of Minas Gerais, northward and then eastward, finally emptying into the Atlantic Ocean along the coasts of the Brazilian states of Alagoas and Sergipe. There are six major dams along its main course, from upstream to downstream: Três Marias, Sobradinho, Luiz Gonzaga (Itaparica), Paulo Afonso complex, Xingó and Paulo Afonso IV (or Apollonius Sales/Moxotó). Among the main reservoirs on the São Francisco River, built for flow regulation and/or hydroelectric power generation, are Três Marias in Minas Gerais; Sobradinho, Paulo Afonso, and Itaparica in Bahia; and Xingó, located on the border between the states of Alagoas and Sergipe (Andrade et al., 2024). Located approximately 440 km from the mouth, the Itaparica Reservoir, situated in the sub‐middle reaches of the São Francisco River, was impounded in 1988 and covers an area of 828 km^2^. In 1992, this reservoir produced 4000 tons of fish. The Xingó Reservoir, located in the lower São Francisco River, was completed in 1994, resulting in the flooding of an area of 60 km^2^. However, fish production data for this reservoir during the studied period are not available (Agostinho et al., 2007). In the unimpounded (free‐flowing) section of the river, large migratory fish species dominate the ichthyofauna, including the spotted sorubim *Pseudoplatystoma corruscans* (Spix and Agassiz, 1829), the zulega *Prochilodus argenteus* (Spix and Agassiz, 1829) and the dorado *Salminus brasiliensis* (Cuvier, 1816). In contrast, the occurrence of migratory species in the reservoirs is sporadic, with the exception of *Prochilodus* spp. In these impounded environments, non‐migratory species such as *Leporinus friderici* (Bloch, 1794) and *P. squamosissimus* (Heckel, 1840) are predominant (Sato & Godinho, 1999).

Numerous studies have employed morphometric characters to describe fish body shape, offering valuable supplementary insights for fisheries management and conservation (Erguden et al., 2009; Fedor & Briony, 2021; Geladakis et al., 2018). Phenotypic variability in body shape, driven by both environmental and genetic factors (Cardinale et al., 2004), has been widely used as a tool for delineating the population structure of various fish species (Ferreira et al., 2024; Hoff, Dias, Zani‐Teixeira, Soeth, & Correia, 2020a; Moreira et al., 2020). As a result, body morphometric data have proven useful for distinguishing the stock structure of commercially exploited fish populations (Allaya et al., 2016; Erguden et al., 2009; Schroeder et al., 2023). In addition to body shape, otolith morphology has emerged as an effective tool for stock identification (Ferreira et al., 2019; Hoff, Dias, Zani‐Teixeira, & Correia, 2020b; Moreira et al., 2019). This methodology has been successfully applied to characterize fish stocks in systems with high gene flow, particularly in the presence of environmental heterogeneity, and to assess intra‐specific geographic variation in response to environmental gradients (Hoff, Dias, Zani‐Teixeira, & Correia, 2020b; Muniz et al., 2021; Schroeder et al., 2021). When used in combination, body morphometric and otolith shape analyses constitute a powerful approach for investigating fish population structure (Rodgveller et al., 2017; Schroeder et al., 2021; Vasconcelos et al., 2017).

The primary objective of this study was to investigate the population structure of *Plagioscion squamosissimus*, a species harvested by three fishers' associations (Colônias de Pescadores) located in Petrolândia (Pernambuco State), Rodelas (Bahia State) and Olhos D'água do Casado (Alagoas State), within the sub‐middle and lower stretches of the São Francisco River basin in the northeastern Brazil. To achieve this, geometric morphometrics analyses of body shape and otolith morphology were applied. Additionally, this study compared alternative methods for detecting variation in both body (truss networking and Procrustes coordinates) and otolith (elliptic Fourier descriptors and wavelet transform coefficients) shape, assessing their discriminatory power in the context of fish stock differentiation. The ultimate goal was to generate insights that support fisheries agencies and decision‐makers in the rational and sustainable management of this resource.

## 2 MATERIALS AND METHODS

### 2.1 Study area

The study area is located in the sub‐middle section of the São Francisco River, encompassing the fluvial stretch between the Itaparica and Xingó reservoirs. This portion of the river flows through parts of the states of Pernambuco, Bahia, Alagoas and Sergipe, and is characterized by a semi‐arid climate, with low rainfall and high evapotranspiration rates, factors that exacerbate local water vulnerability. The predominant biome is the Caatinga, although remnants of riparian forest can still be found in wetter or protected areas. The presence of large‐scale reservoirs has substantially altered the river's natural hydrological regime, contributing to flow regulation but also producing significant impacts on aquatic ecosystems and the human populations that depend directly on the river for their livelihoods (ANA, 2022; CBHRS, 2016).

This stretch of the São Francisco River holds notable socioeconomic importance, particularly for riverside communities engaged in artisanal fishing, small‐scale irrigated agriculture and extractivism. Changes in river dynamics, combined with increasing anthropogenic pressures, including intensive water use for irrigation, unplanned urban expansion and environmental degradation, have compromised aquatic biodiversity and traditional ways of life (Basto et al., 2020; Silva et al., 2018). Additionally, artificial flow regulation disrupts reproductive cycles of native species and sediment distribution, affecting both fish productivity and the fertility of alluvial soils. In this context, the area between Itaparica and Xingó represents a critical zone for understanding the interactions between hydrological regulation, environmental conservation and the sustainability of multiple uses of São Francisco River resources (ANA, 2022; Medeiros et al., 2022).

The Itaparica Reservoir, located in Pernambuco and Bahia, spans approximately 828 km^2^ along a 150‐km stretch of the São Francisco River. It has an average depth of 18–30 m (maximum ~101 m) and a storage capacity of 10.7 billion m^3^. Managed by Companhia Hidroelétrica do São Francisco (CHESF), Itaparica experiences high evaporation rates and operational water level fluctuations of up to 5.5 m. Average discharge is around 820 m^3^ s^−1^, and surface water temperature remains relatively stable at approximately 25 °C. The reservoir supports intensive agricultural activity and fish farming, which contribute to significant nutrient inputs. Surface dissolved oxygen (DO) concentrations range seasonally from 4.6 to 11.7 mg L^−1^, with lower values typically during the dry season. In contrast, the Xingó Reservoir, located downstream across Alagoas, Bahia and Sergipe, covers about 60 km^2^ over a 60‐km stretch, with an average depth of 170 m and a storage capacity of 3.8 billion m^3^. Xingó is also operated by CHESF, with an average discharge of approximately 850 m^3^ s^−1^, and stable surface temperatures around 26 °C. Seasonal DO levels range from 8 to 12 mg L^−1^. The Xingó surrounding area is mainly designated for conservation and tourism, particularly within the Rio São Francisco Natural Monument. Activities in the region focus on navigation, ecotourism, cultural heritage and recreational use, making it a less intensively used reservoir in terms of productive land and water use (Matta et al., 2018; Rossiter et al., 2020; Silva et al., 2018).

### 2.2 Fish sampling

**FIGURE 1:**
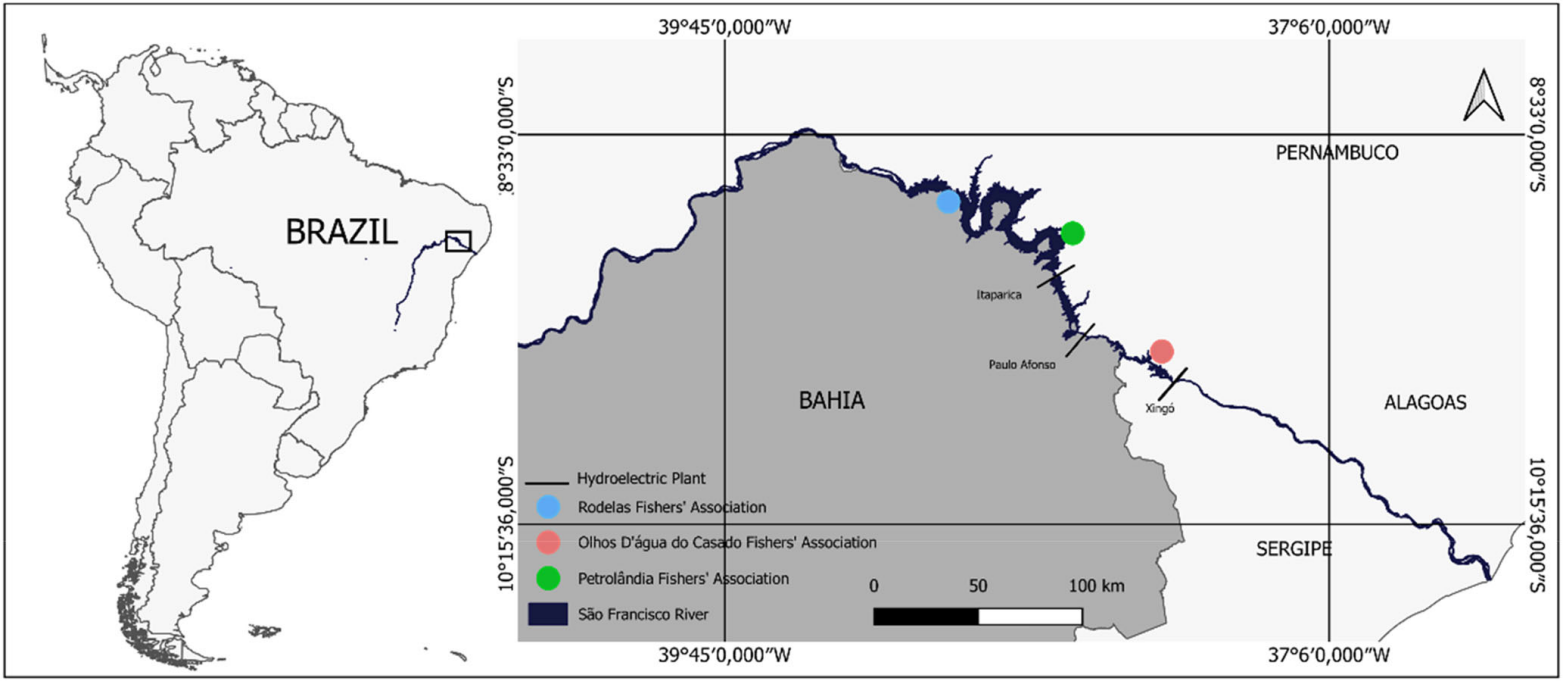
Map showing the three sampling sites of *Plagioscion squamossissimus* caught from September 2023 to March 2024 in the sub‐medium and lower courses of São Francisco River (Rodelas: Bahia State, Petrolândia: Pernambuco State and Olhos D'água do Casado: Alagoas State), in the northeast region of Brazil.

A total of 150 *Plagioscion squamosissimus* individuals were collected from the São Francisco River in northeastern Brazil between September 2023 and March 2024. Sampling was conducted using gills nets at three fishers' associations, with 50 specimens obtained from each site: Rodelas (ROD) in Bahia State and Petrolândia (PET) in Pernambuco State, both located in the sub‐middle section of the river, and Olhos D'água do Casado (OLD) in Alagoas State, situated in the lower section (Figure 1). Fish collection was authorized by the Ethics Committee for Animal Use in Science of the State University of Bahia (protocol no. 01/2017). Immediately after capture, fish were preserved on ice and transported to the laboratory. There, each fish was measured for standard length (SL, to the nearest 0.1 cm), weighed for total mass (FM, to the nearest 0.1 g) and the left side of each specimen was photographed with anatomical landmarks for subsequent body shape analysis (Erguden et al., 2009). Sagittal otoliths were extracted using plastic forceps, rinsed with distilled water to remove organic residues, air‐dried and stored in labelled plastic tubes until analysis. Otolith pairs were distinguished based on the orientation of the acoustic sulcus and rostrum (Secor et al., 1992), with only right sagittae used in the otolith shape analyses. Following evisceration, sex was determined through visual macroscopic examination of gonads (Vazzoler, 1996). All individuals selected for body morphometric and otolith shape analyses were adults, with SL exceeding 19.6 cm, based on the maturity threshold establish to the species (Lima et al., 2019) (Table 1).

**TABLE 1:** Sampling sites, state, river long profiles, geographic coordinates, sample size (*N*), standard length (SL), fish mass (FM) and otolith length (OL) of *Plagioscion squamosissimus* used for body geometric morphometrics and otolith shape analyses.

Sites	State	River long profile	Latitude/longitude	*N*	SL (cm)	FM (g)	OL (mm)
Rodelas	Bahia	Sub‐middle course	8°50′43″S, 38°46′23″W	50	24.2 ± 1.6	237 ± 47.9	15.4 ± 1.2
Petrolândia	Pernambuco	Sub‐middle course	8°59′00″S, 38°13′28″W	50	26.8 ± 2.1	316 ± 71.7	14.4 ± 1.4
Olhos D'água do Casado	Alagoas	Lower course	9°30′06″S, 37°50′01″W	50	27.8 ± 1.8	325 ± 63.8	15.6 ± 1.1

*Note*: Values are expressed as means ± standard errors.

### 2.3 Otolith shape analyses

**FIGURE 2:**
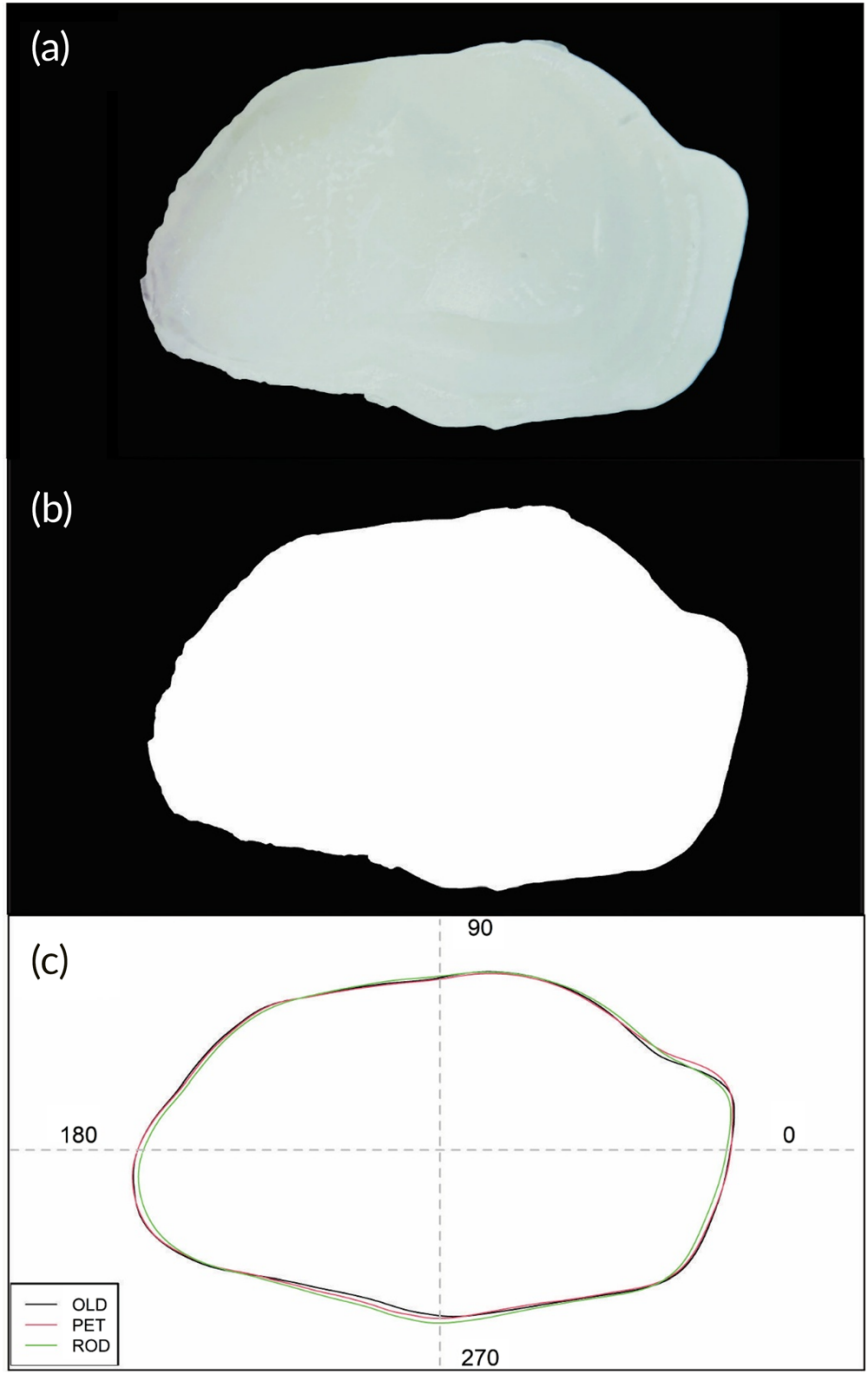
Medial side of a right sagitta from *Plagioscion squamosissimus* for the different sampling sites in the São Francisco river: (a) the original photograph, (b) the binary black and white digital image, and (c) the otolith averaged outline contour for each site. OLD, Olhos D'água do Casado; PET, Petrolândia; ROD, Rodelas.

Digital images of the right sagittal otoliths were captured using a stereomicroscope (EMZ‐13TR; Meiji Techno) equipped with a USB digital camera (SC30; Olympus Corporation). Imaging was performed under reflected light against a dark background at 2× magnification. Each otolith was oriented with the sulcus acusticus facing upward and the rostrum directed to the left (Figure 2a). Care was taken to ensure that the natural curvature of the otolith did not introduce distortions or artefacts that could compromise shape analysis (Volpedo & Vaz‐dos‐Santos, 2015). Orthogonal two‐dimensional images of all 150 otoliths were converted to grayscale and binarized (Figure 2a) using a threshold pixel value of 0.1, implemented via the shapeR package (Libungan & Pálsson, 2015). Otolith outlines were automatically extracted from all images following background removal and image binarization (Figure 2b). To enhance resolution and minimize pixel noise, a weighted moving average was applied across three consecutive coordinate points (Claude, 2008), resulting in smoothed multiple outlines (Libungan & Pálsson, 2015).

Mean otolith outlines were quantified using standardized elliptic Fourier descriptors (EFDs) and wavelet transform coefficients (WTCs) (Figure 2c). To account for potential allometric effects, otolith shape variation was adjusted for size using otolith length, which was recorded during image acquisition. Any EFDs and WTCs showing significant interactions between sampling site and standard length (*p* < 0.05) were automatically excluded from further analysis to minimize confounding effects. To correct for inflated type I error rates due to multiple testing of shape coefficients, a Bonferroni adjustment was applied as a conservative measure to remove residual allometric effects (Libungan & Pálsson, 2015; Sokal & Rohlf, 1995).

For the EFDs, the first four harmonics accounted for 90% of the cumulative power in describing otolith shape, leading to the exclusion of the coefficients c4 and d4. Additionally, since the elliptical Fourier harmonics were normalized to the first harmonic for each otolith, rendering them invariant to otolith size (Kuhl & Giardina, 1982), the first three coefficients (a1, b1 and c1), which were constant across all outlines, were also excluded. This resulted in a final set of 11 EFDs, calculated as follows: (4 harmonics × 4 coefficients) – 3 (a1, b1 and c1) – 2 (c4 and d4) = 11.

Regional differences in otolith outlines were compared using a permutation‐based test, namely a Analysis of Variance with Canonical Correlation Analysis (ANOVA.CCA) on the standardized wavelet descriptors, followed by a constrained analysis of principal coordinates (CAP) (Libungan & Pálsson, 2015; Oksanen et al., 2020; Schroeder et al., 2021).

### 2.4 Body shape analyses

**FIGURE 3:**
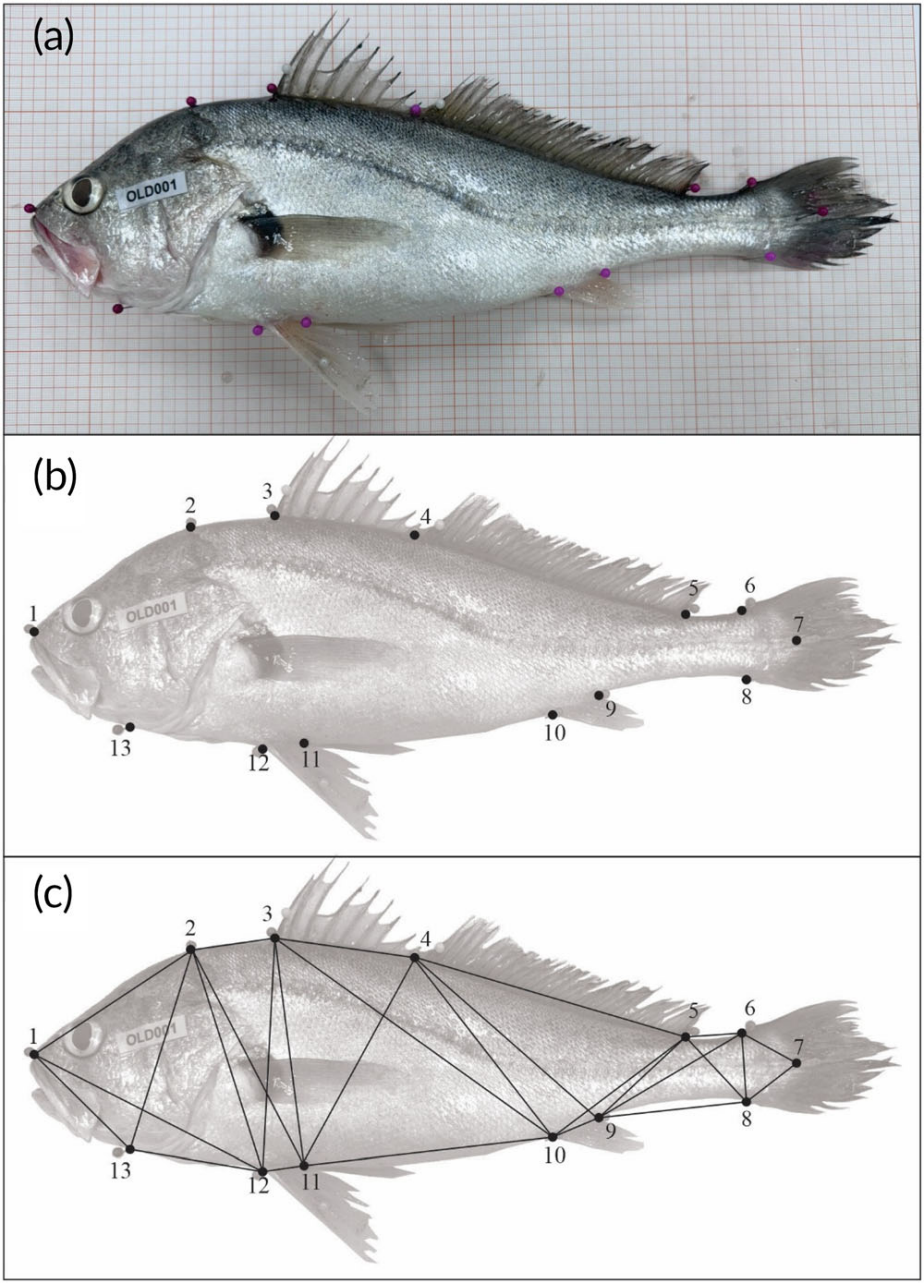
*Plagioscion squamosissimus* photograph from a specimen collected from São Franciso River (standard length of 33.0 cm) in September 2023: (a) illustration with the location of the 13 selected body landmarks (see Table 2 for more details), (b) the generated body morphometric and (c) distances.

Two landmark‐based geometric morphometrics, Procrustes coordinates (PCs) and truss networking (TD), were employed to assess the body shape of *Plagioscion squamosissimus* individuals (Luo, 2024). For both analyses, left lateral photographs of the fish were taken from a fixed distance using a high‐resolution digital camera (12 MP, 3024 × 4032 pixels) and precision scale (10 mm) (Figure 3a) to minimize distortion, following standard guidelines (Muir et al., 2012). A total of 13 anatomical landmarks were identified along the body contour from the lateral view of each fish (Figure 3b,c). The coordinates of homologous landmarks were processed and digitized using tpsUtil Version 1.83 (Rohlf, 2023) and tpsDig Version 2.32 (Rohlf, 2021) software.

The phenotypic variability of *Plagioscion squamosissimus* was assessed through body morphometrics using the 13 anatomical landmarks positioned along the left side of each individual (Figure 3b and Table 2), following protocols outlined in previous studies (Geladakis et al., 2018; Schroeder et al., 2021; Stern et al., 2018). The landmarks were initially subject to generalized Procrustes analysis (GPA) for translation, rotation and scaling across all individuals (Rohlf, 2001; Zelditch et al., 2004). Translation and rotation were achieved through the superimposition method, adjusting for individual inclination and relative position. This adjustment minimized the sum of squared differences between corresponding landmarks, aligning them to a common centroid size (Rohlf, 1999). Shape differences were quantified by comparing PCs derived from GPA (Zelditch et al., 2004). Individual size was estimated using centroid size, calculated as the square root of the sum of squared deviations of landmarks from the centroid point (Rohlf & Cannatella, 1998).

**TABLE 2:** Body landmarks defined along the body contour of *Plagioscion squamosissimus* and morphometric distances used for the body shape analysis.

Body landmark	
Number	Location
1	Anterior tip of snout at upper jaw
2	Most posterior aspect of neurocranium (beginning of scaled nape)
3	Anterior insertion of the first dorsal fin
4	Intersection of the first dorsal fin with the second dorsal fin
5	Posterior insertion of the second dorsal fin
6	Anterior attachment of dorsal membrane from caudal fin
7	Posterior end of the vertebrae column
8	Anterior attachment of ventral membrane from caudal fin
9	Posterior insertion of the anal fin
10	Anterior insertion of the anal fin
11	Posterior insertion of the pelvic fin
12	Anterior insertion of the pelvic fin
13	Posterior most point of the maxillary

Morphometric distances		
Distance	Landmark	Description
D1	1 to 2	Head length
D2	1 to 12	Distance between the anterior part of the head and the anterior portion of the pelvic fin
D3	1 to 13	Maxilla length
D4	2 to 3	Distance from the most posterior aspect of neurocranian to the anterior insertion of the dorsal fin
D5	2 to 11	Distance from the most posterior aspect of neurocranian to the posterior insertion of the pelvic fin
D6	2 to 12	Posterior height of the head
D7	2 to 13	Anterior height of the head
D8	3 to 4	Length of first dorsal fin
D9	3 to 10	Distance between the origin of first dorsal fin and the origin of anal fin
D10	3 to 11	Distance between the origin of first dorsal fin and the posterior insertion of the pelvic fin
D11	3 to 12	Anterior body height
D12	4 to 5	Length of second dorsal fin
D13	4 to 9	Distance between second dorsal fin and posterior insertion of the anal fin
D14	4 to 10	Distance between second dorsal fin and anterior insertion of the anal fin
D15	4 to 11	Distance between the origin of second dorsal fin and the posterior insertion of the pelvic fin
D16	5 to 6	Distance between second dorsal fin and caudal fin
D17	5 to 8	Anterior diagonal of the caudal peduncle
D18	5 to 9	Anterior caudal peduncle height
D19	5 to 10	Distance between second dorsal fin and anterior insertion of the anal fin
D20	6 to 7	Distance between the dorsal insertion of the caudal fin and the posterior end of the vertebrate column
D21	6 to 8	Posterior caudal peduncle height
D22	6 to 9	Posterior diagonal of the caudal peduncle
D23	7 to 8	Distance between the ventral insertion of the caudal fin and the posterior end of the vertebrate column
D24	8 to 9	Distance between anal fin and caudal fin
D25	9 to 10	Length of the anal fin
D26	10 to 11	Distance between posterior insertion of the pelvic fin and anterior insertion of the anal fin
D27	11 to 12	Length of the pelvic fin
D28	12 to 13	Distance from maxilla to pelvic fin

*Note*: For more details, please see the Materials and methods section and Figure 3.

A multivariate regression analysis between body morphometric scores of the dependent variable (PCs) and size (independent variable, SL) was applied to examine the presence of changes in shape related to changes in size (allometry) (Schroeder et al., 2021). The variability in body morphometrics was visualized across the three sampling sites through a principal component analysis for mixed data (PCAmix) performed over the covariance matrix of the PCa (Klingenberg, 2016; Vasconcelos et al., 2017; Zelditch et al., 2004). The scores of the principal component analysis were stored in a new dataset, which was further used to analyse the morphometric variation across sampling sites. To visualize the morphometric variation, a scatter plot of the two first principal components and the logarithm of centroid size (CS) was built (Loy et al., 2001). These scores, also known as relative warps (RWs), are considered free of the effect of allometry (Klingenberg, 2016) and contain more shape information than those of the linear trusses (Bookstein, 1992; Porrini et al., 2015). Squared loadings were calculated for the categorial variable (sampling sites) as correlation ratios between the variable and the principal components. For quantitative variables, squared loadings were computed as the squared correlations between the variable (residuals from the multivariate regression of PCs on CS) and the principal components, with components derived from the *x* and *y* coordinates of each landmark (e.g. LM1X, LM1Y). The correlation between squared loadings for both categorical and numerical variables was used to identify morphometric features associated with site‐specific differentiation (Schroeder et al., 2021).

The residuals from the multivariate regression of the PCs on centroid size (CS) were tested for differences between sampling sites using a one‐way analysis of variance (ANOVA). When significant differences were identified (*p* < 0.05), post hoc comparisons were made using a Tukey test. Additionally, univariate tests were performed to assess significant differences between sampling sites for the first two relative warps (RW1 and RW2), as well as their regression on CS. These tests were conducted using a generalized additive model for location and scale (GAMLSS; Stasinopoulos et al., 2018), with three relationships evaluated: (i) RW2 × RW1, (ii) RW1 × CS and (iii) RW2 × CS.

A standard protocol (Strauss & Bookstein, 1982) was employed to measure 28 linear distances (D) within the box–truss network (Table 2). The relationship between these morphometric distances (D1 to D28) and fish standard length (SL) was tested using a one‐way analysis of covariance (ANCOVA), with SL as the covariate and site as the fixed factor. This analysis revealed significant positive correlations for all distances (*p* < 0.05). To account for the size effect, each distance was corrected using the following transformation to address the positive allometric relationship between the variables (Ferreira et al., 2024): TD = 10ˆ[log(D) − *β*[log(SL) − log(SLmean)]], where TD is the transformed distance, D is the original distance, β is the slope of the regression of log(D) on log(SL), SL is the individual's standard length and SLmean is the overall mean standard length across all sites.

### 2.5 Statistical analysis

Data were tested for normality using the Shapiro–Wilk test (*p* > 0.05) and for homogeneity of variances using Levene's test (*p* > 0.05). Initial statistical analyses included a one‐way ANOVA to assess differences between sexes across sites. Significant differences were observed between males and females for both otolith shape analyses (EFDs and WTCs), but not for the body shape analyses (PCs and TD). One‐way ANOVA was subsequently used to examine univariate differences in otolith (EFDs and WTCs) and body (PCs and TD) shape across sites, with post hoc pairwise comparisons conducted using Tukey's where applicable (*p* < 0.05). Permutational multivariate analysis of variance (PERMANOVA, main and pairwise, if necessary, *p* < 0.05) tests were performed to evaluate multivariate differences among sites for otolith shape analyses and body geometric morphometrics using the Gower distance matrix (Gower, 1996). To further assess classification accuracy, a flexible discriminant function analysis (FDA) was conducted, followed by a jackknifed reclassification matrix (leave‐one‐out cross validation) to determine the percentage of correctly reclassified individuals into their original site, as previously applied in similar studies (Schroeder et al., 2023). Univariate statistical analyses were performed using SigmaPlot 11.0, while multivariate tests were carried out using R 4.3.0 (R Core Team, 2023). A significance level of *α* = 0.05 was adopted and data were presented as mean *±* standard error.

## 3 RESULTS

Otolith shape analysis revealed significant differences between sex in otolith contours (ANOVA.CCA: *df* = 2, *F* = 4.211, *p* = 0.001), although the overall pattern was consistent across sites (Figure S1). Additional analyses conducted separately by sex can be found in Table S1. All shape analyses presented here were performed without separating by sex.

**FIGURE 4:**
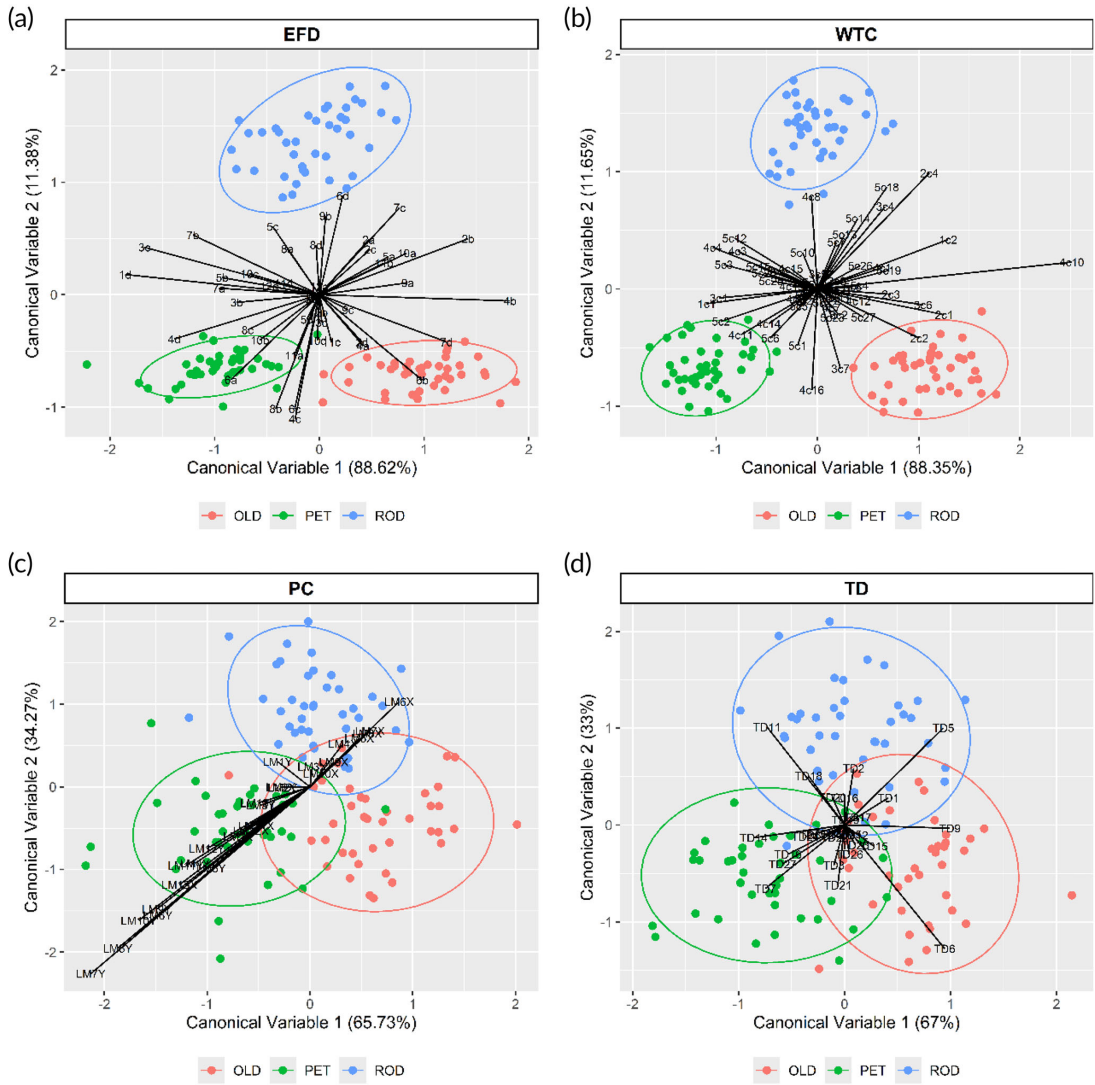
Canonical variable plots showing the differences for otolith contour shape and body geometric morphometrics of *Plagioscion squamosissimus* for the different sampling sites in the São Francisco river: (a) elliptic Fourier descriptors (EFDs), (b) wavelet transform coefficients (WTCs), (c) Procrustes coordinates (PCs) and (d) transformed distances (TDs). Ellipses represent 95% confidence intervals around the data and points represent individual fish. OLD, Olhos D'água do Casado; PET, Petrolândia; ROD, Rodelas.

Regarding the otolith shape analyses, univariate tests indicated significant differences among sites for nearly all elliptical Fourier descriptors (EFDs), except for D2, A3 and B4 (ANOVAs, *df* = 2147, *p* < 0.05; Table 3). Multivariate analysis of otolith shape variables (EFDs) also revealed significant differences among regions (PERMANOVA: pseudo‐*F* = 3.796, *p* < 0.05; Table 4). Furthermore, all pairwise comparisons showed significant differences among sites (pseudo‐*t*‐test, *p <* 0.05). Discriminant function analysis (DFA) indicated no overlap of individuals among sampling sites based on EFDs (Figure 4a). The jackknife matrix based on EFDs presented an overall reclassification success rate of 100% (Table 5).

**TABLE 3:** Results from the univariate statistics regarding the otolith shape analyses (elliptical Fourier descriptors and wavelet transform coefficients) and body geometric morphometrics (Procrustes coordinates and transformed distances) calculated for *Plagioscion squamosissimus* individuals.

	Rodelas	Petrolândia	Olhos D'Água do Casado
Elliptical Fourier descriptor			
D1	18,171.00 ± 2474.00^a^	−7933.52 ± 2474.00^b^	−10,250.08 ± 2474.00^b^
A2	3964.94 ± 2091.00^a^	1904.14 ± 2091.00^a^	−5874.12 ± 2091.00^b^
B2	1931.86 ± 2066.00^a^	3929.94 ± 2066.00^a^	−5860.82 ± 2066.00^b^
C2	4573.86 ± 1480.00^a^	−3152.62 ± 1480.00^b^	−1417.92 ± 1480.00^b^
B3	310.80 ± 859.00^ab^	2152.92 ± 859.00^a^	−2460.38 ± 859.00^b^
C3	−605.34 ± 850.00^ab^	2155.10 ± 850.00^a^	−1548.64 ± 850.00^b^
A4	−813.98 ± 699.00^a^	−1143.86 ± 699.00^a^	1954.08 ± 699.00^b^
Wavelet transform coefficient			
W1	0.5678 ± 0.0172^a^	0.4731 ± 0.0572^a,b^	0.4001 ± 0.0337^b^
W2	0.7853 ± 0.0234^a^	0.6390 ± 0.0610^b^	0.7303 ± 0.0294^a,b^
W3	2.4919 ± 0.0141^a^	2.4772 ± 0.0538^a^	2.7296 ± 0.0157^b^
W4	0.2329 ± 0.0063^a^	0.4162 ± 0.0344^b^	0.5407 ± 0.0228^c^
W5	0.3441 ± 0.0142^a^	0.3068 ± 0.0192^a^	0.4148 ± 0.0127^b^
W6	0.8135 ± 0.0130^a^	0.5337 ± 0.0149^b^	0.5031 ± 0.0136^b^
W10	0.4328 ± 0.0117^a^	0.3235 ± 0.0124^b^	0.3626 ± 0.0103^c^
W12	0.5564 ± 0.0117^a,b^	0.5868 ± 0.0153^a^	0.5236 ± 0.0121^b^
W13	0.2732 ± 0.0065^a^	0.3158 ± 0.0057^b^	0.3388 ± 0.0071^c^
W14	0.2613 ± 0.0080^a^	0.2375 ± 0.0089^a,b^	0.2284 ± 0.0068^b^
W21	0.2195 ± 0.0025^a^	0.2346 ± 0.0050^b^	0.2328 ± 0.0048^a,b^
W22	0.3775 ± 0.0046^a^	0.3504 ± 0.0074^b^	0.3631 ± 0.0070^a,b^
W24	0.3958 ± 0.0072^a^	0.3550 ± 0.0098^b^	0.4197 ± 0.0095^a^
W25	0.2631 ± 0.0057^a^	0.2441 ± 0.0074^a,b^	0.2296 ± 0.0058^b^
W30	0.1204 ± 0.0024^a^	0.1602 ± 0.0041^b^	0.1578 ± 0.0032^b^
W33	0.2263 ± 0.0018^a^	0.2293 ± 0.0027^a^	0.2035 ± 0.0025^b^
W36	0.1215 ± 0.0012^a^	0.1383 ± 0.0015^a^	0.1364 ± 0.0010^b^
W37	0.1678 ± 0.0019^a^	0.1437 ± 0.0021^b^	0.1507 ± 0.0012^c^
W42	0.1281 ± 0.0007^a^	0.1247 ± 0.0011^a^	0.1107 ± 0.0010^b^
W44	0.1318 ± 0.0016^a,b^	0.1389 ± 0.0029^b^	0.1288 ± 0.0023^a^
W45	0.2404 ± 0.0031^a,b^	0.2321 ± 0.0044^b^	0.2516 ± 0.0042^a^
W46	0.1914 ± 0.0035^a^	0.1864 ± 0.0035^a,b^	0.1767 ± 0.0035^b^
W48	0.2131 ± 0.0022^a^	0.1824 ± 0.0009^a^	0.1819 ± 0.0016^b^
W49	0.1222 ± 0.0008^a^	0.1224 ± 0.0007^a^	0.1291 ± 0.0010^b^
W50	0.1381 ± 0.0010^a^	0.1383 ± 0.0014^a^	0.1275 ± 0.0008^b^
W53	0.1511 ± 0.0012^a^	0.1466 ± 0.0013^a,b^	0.1428 ± 0.0017^b^
Procrustes coordinate			
LM1Y	−0.0073 ± 0.0012^a^	−0.0026 ± 0.0015^b^	−0.0068 ± 0.0015^a,b^
LM2Y	0.1160 ± 0.0006^a^	0.1110 ± 0.0009^b^	0.1137 ± 0.0006^a^
LM3Y	0.1379 ± 0.0009^a^	0.1319 ± 0.0009^b^	0.1348 ± 0.0009^a,b^
LM7X	0.3716 ± 0.0006^a^	0.3748 ± 0.0007^b^	0.3755 ± 0.0006^b^
LM9Y	−0.0521 ± 0.0008^a^	−0.0503 ± 0.0007^a,b^	−0.0493 ± 0.0008^b^
LM10Y	−0.0760 ± 0.0008^a^	−0.0738 ± 0.00083^a,b^	−0.0718 ± 0.0010^b^
LM11Y	−0.1026 ± 0.0009^a^	−0.1002 ± 0.0011^a,b^	−0.0981 ± 0.0009^b^
LM12X	−0.1805 ± 0.0008^a^	−0.1824 ± 0.0010^a,b^	−0.1848 ± 0.0010^b^
LM12Y	−0.1109 ± 0.0008^a^	−0.1075 ± 0.0010^b^	−0.1058 ± 0.0008^b^
LM13X	−0.3320 ± 0.0012^a^	−0.3397 ± 0.0014^b^	−0.3278 ± 0.0015^a^
LM13Y	−0.0832 ± 0.0007^a^	−0.0874 ± 0.0008^b^	−0.0885 ± 0.0008^b^
Transformed distances			
TD1	58.64 ± 0.68^a^	60.18 ± 0.68^ab^	61.38 ± 0.68^b^
TD3	34.60 ± 0.47^a^	35.68 ± 0.47^a^	37.90 ± 0.47^b^
TD6	75.48 ± 0.84^a^	77.50 ± 0.84^ab^	78.78 ± 0.84^b^
TD7	63.90 ± 0.61^a^	67.96 ± 0.61^b^	67.50 ± 0.61^b^
TD8	48.86 ± 0.48^a^	50.24 ± 0.48^ab^	50.80 ± 0.48^b^
TD9	114.70 ± 0.80^a^	116.80 ± 0.80^ab^	118.16 ± 0.80^b^
TD12	93.28 ± 0.84^a^	97.50 ± 0.84^b^	97.96 ± 0.84^b^
TD13	84.00 ± 0.68^a^	87.00 ± 0.68^b^	87.00 ± 0.68^b^
TD14	79.14 ± 0.70^a^	82.24 ± 0.70^b^	82.60 ± 0.70^b^
TD16	22.46 ± 0.46^a^	24.62 ± 0.46^b^	24.58 ± 0.46^b^
TD17	31.28 ± 0.41^a^	31.62 ± 0.41^ab^	32.64 ± 0.41^b^
TD19	55.60 ± 0.82^a^	61.20 ± 0.82^b^	61.30 ± 0.82^b^
TD20	19.84 ± 0.28^a^	20.90 ± 0.28^b^	21.04 ± 0.28^b^
TD22	57.46 ± 0.61^a^	59.72 ± 0.61^ab^	59.02 ± 0.61^b^
TD23	19.58 ± 0.25^a^	20.52 ± 0.25^a^	19.68 ± 0.25^b^
TD25	17.84 ± 0.25^a^	17.12 ± 0.25^b^	15.66 ± 0.25^b^
TD28	47.52 ± 0.61^a^	50.34 ± 0.61^a^	45.78 ± 0.61^b^

*Note*: Variables showing different letters indicate that significant regional differences exist (*p* < 0.05) (one‐way ANOVA, followed by Tukey post hoc tests). Values are expressed as means ± standard errors. Variables without significant differences (one‐way ANOVA, *p* > 0.05) are not shown.

**TABLE 4:** Main and pairwise PERMANOVA comparisons for the otolith shape analyses (elliptic Fourier descriptors and wavelet transform coefficients) and body geometric morphometrics (Procrustes coordinates and transformed distances) among the three *Plagioscion squamosissimus* sampling sites.

Main PERMANOVA	Elliptic Fourier descriptor					Wavelet transform coefficient				Pr (>*F*)
	*df*	SSq	*R* ^2^	*F*	Pr (>*F*)	*df*	SSq	*R* ^2^	*F*	
Region	2	0.267	0.049	3.796	0.0001	2	0.047	0.175	15.540	0.0009
Residual	147	5.171	0.937			146	0.223	0.824		
Total	149	5.438	1			148	0.270	1		

Pairwise PERMANOVA	*df*	SSq	*F*. Model	*R* ^2^	*p*. value	*p*. adjusted	*df*	SSq	F. Model	*R* ^2^	*p*. value	*p*. adjusted
Olhos D'água do Casado vs. Petrolândia	1	0.228	2.181	0.021	0.026	0.078	1	0.012	6.796	0.065	0.001	0.003
Olhos D'água do Casado vs. Rodelas	1	0.728	7.010	0.066	0.001	0.003	1	0.036	33.802	0.256	0.001	0.003
Petrolândia vs. Rodelas	1	0.313	3.199	0.031	0.001	0.003	1	0.022	13.375	0.121	0.001	0.003

Main PERMANOVA	Procrustes coordinate					Transformed distance				
	*df*	SSq	*R* ^2^	*F*	Pr(>*F*)	*df*	SSq	*R* ^2^	*F*	Pr (>*F*)
Region	2	0.678	0.069	17.392	0.0009	2	0.00	0.069	5.524	0.0001
Residual	147	2.867	0.808			147	0.033	0.930		
Total	149	3.546	1			149	0.035	1		

Pairwise PERMANOVA	*df*	SSq	F. Model	*R* ^2^	*p*. value	*p*. adjusted	*df*	SSq	F. Model	*R* ^2^	*p*. value	*p*. adjusted
Olhos D'água do Casado vs. Petrolândia	1	0.056	2.431	0.024	0.047	0.141	1	0.001	1.787	0.017	0.127	0.381
Olhos D'água do Casado vs. Rodelas	1	0.640	27.724	0.220	0.001	0.003	1	0.006	9.163	0.085	0.001	0.003
Petrolândia vs. Rodelas	1	0.495	21.819	0.182	0.001	0.003	1	0.004	5.536	0.053	0.003	0.090

**TABLE 5:** Summary of the percentage of correct reclassification using the training base set following a flexible discriminant analysis for otolith‐shape elliptical Fourier descriptors, wavelet transform coefficients and the body‐shape morphometrics Procrustes coordinates and transformed distances calculated for *Plagioscion squamosissimus* individuals.

Original location	Predicted location						Total	Re‐classification	
	Training set			Test set				% of correct re‐allocation	% of overall re‐allocation
	OLD	PET	ROD	OLD	PET	ROD			
Elliptical Fourier descriptors									
OLD	40	0	0	10	0	0	50	100	
PET	0	40	0	0	10	0	50	100	100
ROD	0	0	40	0	0	10	50	100	
Wavelet transform coefficients									
OLD	40	0	0	10	0	0	50	100	
PET	0	40	0	0	10	0	50	100	100
ROD	0	0	40	0	0	10	50	100	
Procrustes coordinates									
OLD	36	1	3	10	0	0	50	92	
PET	1	39	0	0	10	0	50	98	94
ROD	2	0	38	0	0	10	50	93	
Transformed distances									
OLD	36	3	1	10	0	0	50	88	
PET	4	35	1	0	10	0	50	90	91
ROD	1	1	38	0	0	10	50	95	

Abbreviations: OLD, Olhos D'água do Casado; PET, Petrolândia; ROD, Rodelas.

The reconstruction of the otolith outline was more accurately described by WTCs compared to EFDs (Figure 4). A permutation‐based test revealed significant differences among fish collected from different sampling sites (ANOVA.CCA: *df* = 2, *F* = 6.089, *p* < 0.001). The jackknife matrix based on WTCs demonstrated an overall reclassification success rate of 100% (Table 5). The FDA showed a clear separation of individuals among sites (Figure 4b).

Body morphometrics (PCs and TD) showed no significant differences between sex (ANOVA: *df* = 2, *F* = 1.037, *p* = 0.162). Eleven out of the 26 PCs evaluated in univariate tests exhibited significant differences among the three sampling sites (ANOVAs and Tukey post hoc tests, *p* < 0.05; Table 3). Multivariate regression of body morphometrics on centroid size was significant (9999 random permutations, *p* < 0.001). The PCAmix analysis revealed moderate explanatory power for the first two relative warps. Squared correlations indicated that two variables, LM2Y and LM7X, were most strongly associated with the second relative warp and the categorical variable (sampling sites: REGION) (Figure 4c). Seven other landmarks (LM1Y, LM11Y, LM13Y, LM12X, LM10Y, LM13X and LM9X) were more closely associated with the first relative warp, while two landmarks (LM3Y and LM12Y) were related to other relative warps (Figure 4c, Table 2 and Figure S1). These associations primarily involved landmarks located on the head, anal, caudal and pelvic fins (Table 2).

Multivariate analysis of these variables revealed significant differences among sampling sites (MAIN.PERMANOVA: *df* = 2, *F* = 6.677, *p* = <0.001). Pairwise comparisons showed that fish from OLD and PET exhibited similar body morphometrics (PAIRWISE.PERMANOVA: *df* = 1, *F* = 2.969, *p* = 0.066), whereas fish from ROD differed significantly from both OLD (PAIRWISE.PERMANOVA: *df* = 1, *F* = 5.862, *p* = 0.003) and PET (PAIRWISE.PERMANOVA: *df* = 1, *F* = 3.962, *p* = 0.003).

GAMLSS showed significant differences among sampling sites. RW1 regressed on CS showed no significant differences, while significant differences were observed for RW2 regressed on CS (GAMLSS: standard error = 0.292, *t* value = −8.077, *p* < 0.001), and for RW2 regressed on RW1 (GAMLSS: standard error = 0.259, *t* value = −9.850, *p* < 0.001) (Figure S2).

The jackknife matrix based on PC scores showed an overall reclassification success rate of 94% (Table 5), with highest reclassification accuracy observed for PET (98%), followed by ROD (93%) and OLD (92%) (Table 5).

Univariate tests showed significant differences in body morphometrics among the sites for 17 out of 28 TD (ANOVAs and Tukey post hoc tests, *p* < 0.05; Table 3). However, none of the measurements showed significant differences among all three sampling sites. A total of 39.2% of the TD (e.g. TD2, TD4, TD5, TD10, TD11, TD15, TD18, TD21, TD24, TD26 and TD27) did not differentiate among sites. In contrast, 28.6% of the data (e.g. TD7, TD12, TD13, TD14, TD16, TD19, TD20 and TD25) differentiated between ROD and the remaining sites, while 10.7% (e.g. TD3, TD23 and TD28) distinguished OLD from the other sites. Additionally, 23.1% (e.g. TD1, TD6, TD8, TD9, TD16 and TD20) exhibited differences between ROD and OLD, with no differences observed between ROD and PET. The jackknife matrix used based on TD showed an overall reclassification success rate of 91% (Table 5). The best classification rate was obtained for ROD (95%), followed by PET (90%) and OLD (88%) (Table 5).

The multivariate analysis using TD also revealed significant differences among the three sites (MAIN.PERMANOVA: *p* < 0.05; Table 4) and pairwise tests showed significant differences between ROD and OLD (pseudo‐*t*‐test, *p* < 0.05; Table 4). The FDA plot (Figure 4d) showed a small overlap of individuals among the sampling sites based on the truss network.

## 4 DISCUSSION

Genetic factors and environmental influences are two primary determinants of otolith morphogenesis (Vignon & Morat, 2010). As a result, otolith morphology can show geographic variation within species, which can serve as a phenotypic tool for the identification and delineation of fish stocks (Cardinale et al., 2004; Cadrin et al., 2014; Ferreira et al., 2024). The utility of otolith shape analysis for studying population structure has been well‐established in numerous fish species, particularly marine species (Moreira et al., 2019; Muniz et al., 2021; Schroeder et al., 2021). However, the successful application of otolith shape analysis has also been documented for freshwater and migratory fish species. For instance, a single population of pirarucu (*Arapaima gigas*) was differentiated across sub‐basins of the Mamoré and Guaporé rivers, the primary tributaries on the right bank of the Madeira River in Amazonia (Catâneo et al., 2022; Mereles et al., 2024). Additionally, the morphology of sagittal otoliths has been effectively used to identify a new species of *Astronotus* in the Orinoco River and Gulf of Paria basins in northern South America (Lozano et al., 2022). Sagittal otolith morphology has also proven useful in establishing connectivity patterns between individuals of the Lebranche mullet (*Mugil liza*) sampled in Argentinean waters and those landed in southern Brazil (Schroeder et al., 2023).

The otolith shape signatures presented herein demonstrated regional phenotypic variation in *P. squamosissimus* individuals from the different sites examined in this study. These phenotypic differences may be attributed to the natural environmental conditions experienced by the fish, as well as to the environmental disruptions caused by the presence of hydroelectric dams along the water body. Variations in flooded area and average water depth are likely among several environmental factors influencing the observed differences. Other variables such as light availability, temperature, flow rate and water quality also differ between reservoirs and may contribute to these effects, although they were not directly tested in this study (Sato & Godinho, 2003; Ferreira et al., 2021). Such environmental conditions can influence various fish characteristics, including feeding, growth and behaviour, which may, in turn, lead to variations in otolith shape (Barbarossa et al., 2020; Caldas et al., 2023; Hauser et al., 2024). The observed variations in otolith shape between fish groups indicate distinct environmental histories, suggesting that otolith shape could serve as a reliable indicator for stock discrimination (Kerr & Campana, 2014; Nazir & Khan, 2021; Volpedo & Vaz‐dos‐Santos, 2015). Furthermore, visual inspection of the FDA plots revealed the presence of three distinct clusters. This separation appeared to be primarily driven by variables A2, B2, B3, C3, A4 and B4 in the case of the EFD, and by variables W1, W2, W3, W4, W5, W6, W10, W12, W13, W14, W21, W22, W24, W25, W30, W33, W36, W37, W42, W44, W45, W46, W48, W49, W50 and W53 in the case of the WTC.

The observed differences in otolith shape among sites may be attributed to the adaptive capacity of the species to respond to environmental changes or habitat alterations (Begossi & Silvano, 2008; Dutra et al., 2023; Hallwass et al., 2013). Notably, hydrological and bathymetry differences are known to exist among the study sites (Sato & Godinho, 2003). *Plagioscion squamosissimus* appears to be resilient to river impoundment, as reported in studies conducted in reservoirs of the Paraná River basin (Dutra et al., 2023; Hallwass et al., 2013), which may reflect its ability to adapt to changes in environmental conditions or shifts in dietary preferences (Almeida et al., 1997; Hahn et al., 1998; Bennemann et al., 2006). Similarly, otolith shape in *Pseudoplatystoma metaense* from the Orinoco River has been linked to differences in growth rate, life‐history traits and habitat use among populations (Pérez & Fabré, 2013). Furthermore, behavioural activities such as swimming, feeding and reproduction may influence otolith structure (Lychakov & Rebane, 2000; Mereles et al., 2021; Mérigot et al., 2007). Sex‐related differences in otolith shape may suggest secondary sexual dimorphism (Parmentier et al., 2018; Tuset et al., 2016; Vaux et al., 2019), although current evidence does not support the existence of spatial segregation between sexes (Palazzo et al., 2022; Sion et al., 2020; Tuset et al., 2014).

Body geometric morphometrics based on anatomical landmarks can be an effective tool for spatially differentiating exploited fish species (Ferreira et al., 2024; Kaouèche et al., 2017; Moreira et al., 2019). In the present study, body morphometric analysis revealed significant differences among groups. Multivariate analyses demonstrated a clear separation among samples from Petrolândia, Olhos D'Água do Casado and Rodelas. Notably, the distance between the anterior insertion of the first dorsal fin and the anterior insertion of the anal fin was greater in specimens from Olhos D'água do Casado. All three sampling groups exhibited similar body height. However, head length was greater in fish from Olhos D'água do Casado and Petrolândia, while mouth length was largest in individuals from Olhos D'água do Casado. In fish, including *P. squamosissimus*, mouth length is typically associated with the size of consumed prey items, their relative abundance and the level of difficulty in accessing their habitats (Bozza & Hahn, 2010; Costa et al., 2009; Ferreira Filho et al., 2014).

The observed differences in the body geometric morphometrics of *P. squamosissimus* among these regions may be related to the structural conditions of the studied environment, resulting from the hydroelectric dam structures, that caused changes in the area and depth, among other variables. The sub‐middle stretch of the São Francisco River in Petrolândia and Rodelas represents a transitional environment between a natural river and the formation of a hydroelectric plant lake. In contrast, the lower São Francisco River stretch at Olhos D'agua do Casado is exclusively a hydroelectric plant lake (CHESF, 2021). Several studies have shown significant changes in fish communities after the construction of dams, especially after some degree of stabilization in the environmental conditions (Agostinho et al., 2008; Carvalho & Araújo, 2024; Winemiller et al., 2016). The downstream impacts of the reservoirs appear to be as, or more, important than those upstream due to changes in the seasonal flood cycle (flow control). These effects are more relevant when reservoirs are built in cascades (Agostinho et al., 2008, Fitzgerald et al., 2018; Carvalho et al., 2024). As a result, the hydrological connectivity between environments is considerably modified in space and time, and the redistribution of the flood regime has several direct and indirect effects on fish populations (Liermann et al., 2012; Lujan et al., 2013; Simonovi et al., 2021).

The observed differences in the body geometric morphometrics characteristics of *P. squamossissimus* across the studied sites may be related with variations in feeding strategies, as has been observed for other fish species (Agostinho et al., 2008; Ferreira Filho et al., 2014; Geladakis et al., 2018). The availability and heterogeneity of food resources in aquatic environments are key factors influencing the distribution and structure of fish assemblages (Casatti, 2003; Pelicice et al., 2005; Smith et al., 2003). Notably, few species are capable of persisting in deep, pelagic environments, which often become inhospitable due to low structural complexity and limited resources (Agostinho et al., 2007). Exceptions include some catfish species and the non‐native croaker, *P. squamosissimus*, which are frequently captured in the deeper strata of reservoirs (Agostinho et al., 2008). This species has demonstrated a remarkable capacity to adapt to lacustrine environments, as also observed in the upper Paraná River basin in a study involving approximately 220 fish species, where *P. squamosissimus and Hypophthalmus edentatus* exhibited the highest adaptability (Gomes & Miranda, 2001). Such adaptability is particularly evident in areas subject to anthropogenic influences, including reservoir systems (Lima Jr et al., 2018; Queiroz‐Sousa et al., 2018; Vitule et al., 2009). Effective management of *P*. *squamosissimus* requires prior evaluation of the exploited stocks to ensure the sustainability of fisheries and the conservation of biodiversity in freshwater ecosystems (González & Márquez, 2020; Khan & Khan, 2017; Teixeira et al., 2002).

Both otolith shape analysis and body geometric morphometrics proved effective in identifying discrete population units of *P. squamosissimus* within the study region. A similar finding was reported for *Arapaina gigas* in the upper Madeira River (Mereles et al., 2024). In a related study on a species of the genus *Cichla* (Cichliformes: Cichlidae), otolith morphology was shown to be influenced by multiple factors that are often complex and difficult to interpret, as they may arise from a variety of processes and interactions occurring throughout a fish's life history, including ontogenetic development, adaptive responses, biogeographic patterns and phylogenetic heritage (McLachlan & Ladle, 2011; Mereles et al., 2021; Tuset et al., 2016). While the body and otolith shape differences between sites are clearly supported by the hereby data, the underlying causes, whether genetic, environmental, or a combination of both, were not directly investigated in this study. This implies that further research is needed to clarify the roles of abiotic and biotic factors in shaping the population structure of *P. squamosissimus* within the São Francisco River basin.

## AUTHOR CONTRIBUTIONS

**Fabricio de Lima Freitas:** Writing–original draft, resources, investigation, methodology and formal analysis. **Natan Silva Pereira:** Writing – review and editing, supervision, investigation and resources. **Barros Pinheiro:** Writing – review and editing, writing – original draft, resources and investigation. **Rafael Schroeder:** Writing – review and editing, formal analysis, methodology and investigation. **Alberto Teodorico Correia:** Supervision, methodology, formal analysis, investigation, conceptualization, writing – review and editing.

## Supporting information


**DATA S1.** Supporting information.
