# Supplementary material for: Assessing the population structure of Plagioscion squamosissimus (Teleostei, Perciformes, Sciaenidae) from the São Francisco River, Bahia, Brazil, using body morphology and otolith shape signatures
Source: J Fish Biol. 2025 Sep 11;108(1):69–85. doi: 10.1111/jfb.70221 (PMC13033955; doi:10.1111/jfb.70221)
Supplement: Supplementary file 1 — DATA S1. Supporting information. [file JFB-108-69-s001.docx]

**Assessing the population structure of *Plagioscion squamosissimus* from the São Francisco River, Bahia, Brazil, using body morphology and otolith shape signatures**

Freitas, F.L.^1^, Pereira, N.S.^2^, Pinheiro, P.B.^3^, Schroeder, R.^4^, Correia, A.T.^5,6,*^

*^1^ Department of Technology and Social Science (PPGEcoH/DTCS), State University of Bahia (UNEB), Juazeiro, Brazil.*

*^2^ Department of Exact and Earth Science (PGQA/DCET), State University of Bahia (UNEB), Salvador, Brazil.*

*^3^ Department of Education (LEAQUA/DEDC), State University of Bahia (UNEB), Paulo Afonso, Brazil.*

*^4^ Laboratório de Estudos Marinhos Aplicados, Escola Politécnica, Universidade do Vale do Itajaí (UNIVALI), Itajaí, Brazil.*

*^5^ Interdisciplinary Centre of Marine and Environmental Research (CIIMAR/CIMAR), Terminal de Cruzeiros do Porto de Leixões, Avenida General Norton de Matos S/N, 4450-208 Matosinhos,Portugal.*

*^6^ Department of Aquatic Production (DPA), School of Medicine and Biochemical Sciences (ICBAS), University of Porto (UP), 4050-313 Porto, Portugal*

*Corresponding author: [atcorreia@icbas.up.pt](mailto:atcorreia@icbas.up.pt)

**Supplementary material**


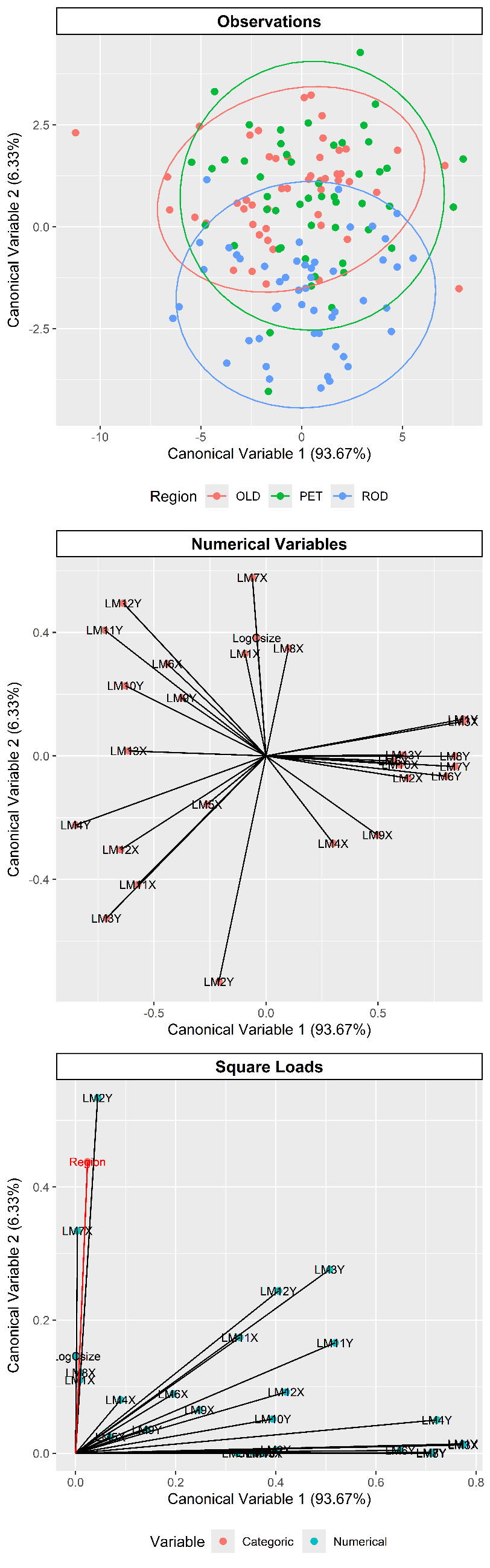


Fig. S1 Principal Components Analysis of mixed data aimed to visualize a possible difference in body morphometric performed over the variance–covariance matrix of the Procrustes coordinates of *Plagioscion squamossissimus* collected in the three-sampling location (Rodelas – Ba, Petrolândia – PE, Olhos D’água do Casado – AL) in the sub medium e low São Francisco River from September 2023 to March 2024. The upper figure represents the component map with factor scores of numerical variables. The lower figure represents the squared loadings of all variables. The red arrow represents the categorical variable (sampling areas).

Table S1. Leave-one-out re-classification matrix of *Plagioscion squamossissimus* based in otolith standardized coefficients of *Plagioscion squamossissimus* collected in the three-sampling location (Rodelas – Ba, Petrolândia – PE, Olhos D’água do Casado – AL) in the sub medium e low São Francisco River from September 2023 to March 2024.

| Sex | Method | Original area | Predicted Location | | | | | | | Re-classification | |
| --- | --- | --- | --- | --- | --- | --- | --- | --- | --- | --- | --- |
|  |  |  | Training set | | | Test set | | | Total | % correct re-classification | % overall re-allocation |
|  |  |  | OLD | PET | ROD | OLD | PET | ROD |  |  |  |
| Males | Wavelets | OLD | 11 | 0 | 0 | 39 | 0 | 0 |  | 100 |  |
|  |  | PET | 0 | 22 | 0 | 0 | 28 | 0 | 50 | 100 | 100 |
|  |  | ROD | 0 | 0 | 36 | 0 | 0 | 14 |  | 100 |  |
|  |  |  |  |  |  |  |  |  |  |  |  |
|  | Fourier | OLD | 10 | 1 | 0 | 39 | 0 | 0 |  | 91 |  |
|  |  | PET | 1 | 21 | 0 | 0 | 28 | 0 | 50 | 95 | 97 |
|  |  | ROD | 0 | 0 | 36 | 0 | 0 | 14 |  | 100 |  |
|  |  |  |  |  |  |  |  |  |  |  |  |
| Females | Wavelets | OLD | 30 | 1 | 0 | 19 | 0 | 0 |  | 100 |  |
|  |  | PET | 0 | 19 | 0 | 0 | 31 | 0 | 50 | 100 | 100 |
|  |  | ROD | 0 | 0 | 5 | 0 | 0 | 45 |  | 100 |  |
|  |  |  |  |  |  |  |  |  |  |  |  |
|  | Fourier | OLD | 30 | 0 | 0 | 20 | 0 | 0 |  | 100 |  |
|  |  | PET | 0 | 19 | 0 | 0 | 31 | 0 | 50 | 100 | 100 |
|  |  | ROD | 0 | 0 | 5 | 0 | 0 | 45 |  | 100 |  |

| 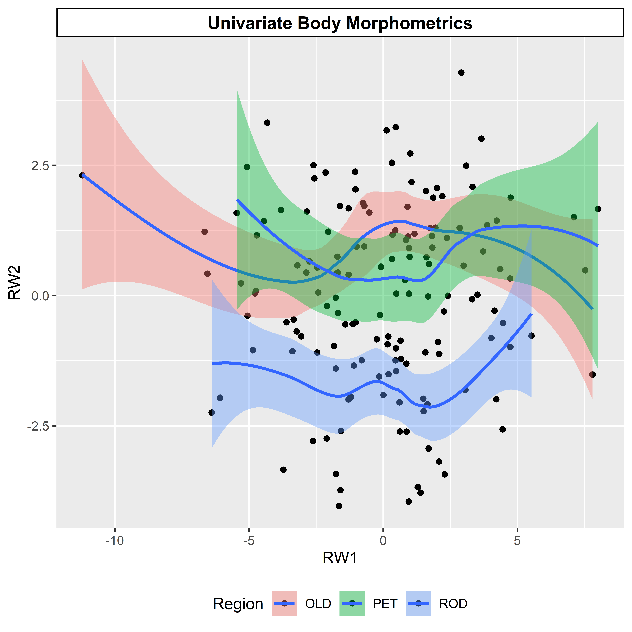 |
| --- |
| 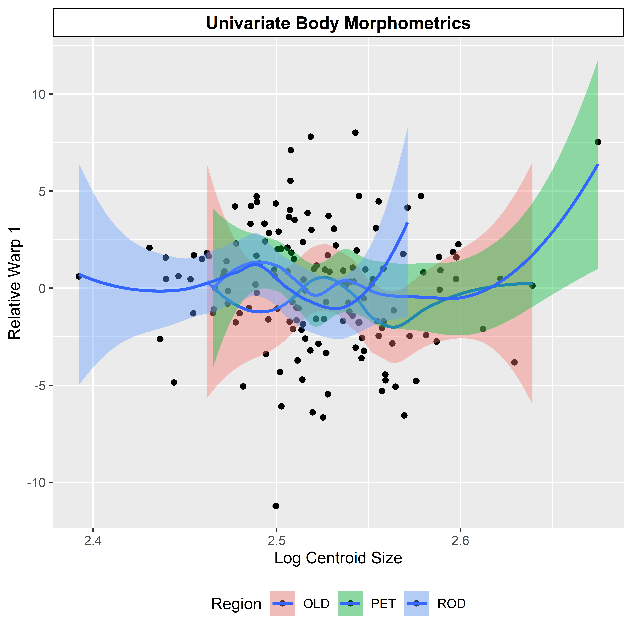 |
| 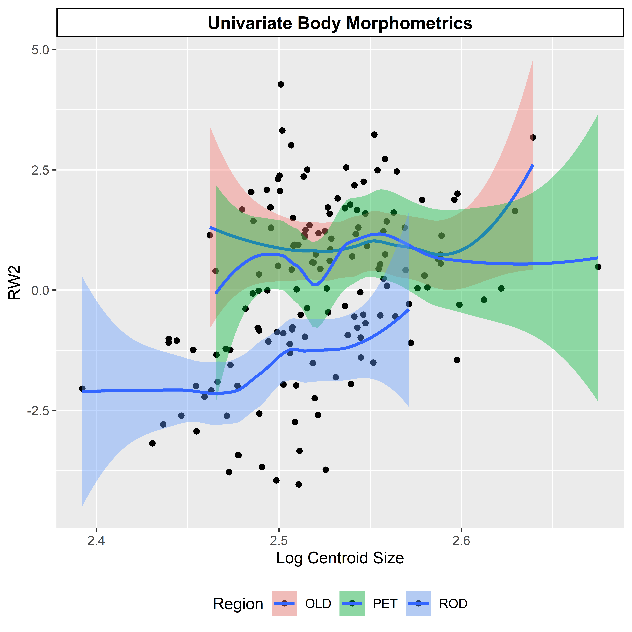 |

Fig. S2 Generalized additive models for location and scale evaluated the existence of significant differences in body morphometrics described from the relative warp (RW) 1 and 2 and their regression on the logarithm value of centroid size (CS) of *Plagioscion squamossissimus* collected in the three-sampling location (Rodelas – Ba, Petrolândia – PE, Olhos D’água do Casado – AL) in the sub medium e low São Francisco River from September 2023 to March 2024 in 3 combinations: RW2xRW1, RW1xCS, and RW2xCS.


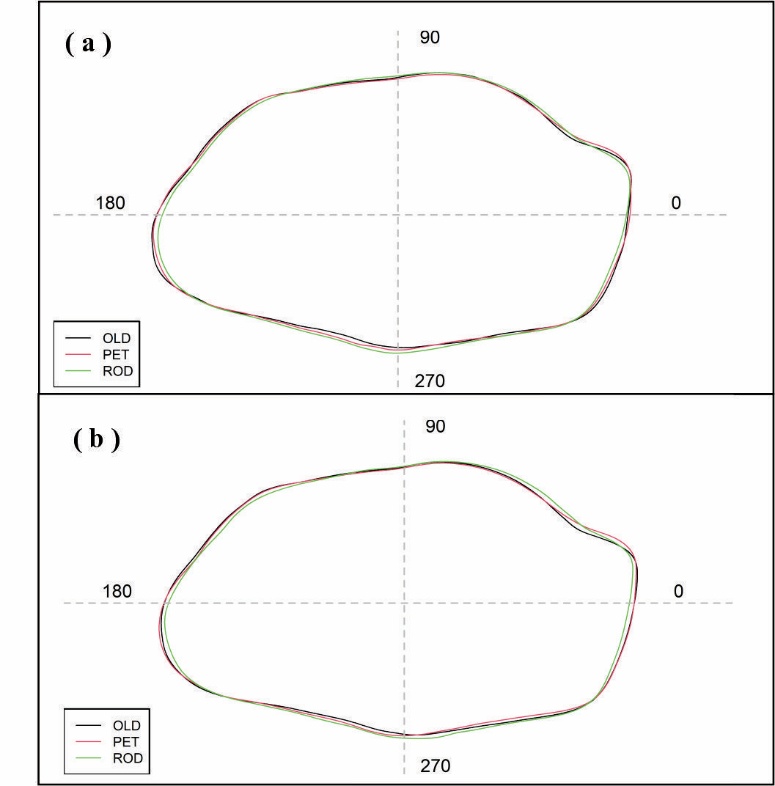


Fig. S3 Medial side of a right sagitta from *Plagioscion squamosissimus* for the different sampling sites in the São Francisco river (Olhos D’água do Casado: OLD, Petrolândia: PET and Rodelas: ROD), showing (a) the otolith averaged outline contour of the males for each site and (b) the otolith averaged outline contour of the females for each site.
